# Engineered Glibenclamide-Loaded Nanovectors Hamper Inflammasome Activation in an Ex Vivo Alzheimer’s Disease Model—A Novel Potential Therapy for Neuroinflammation: A Pilot Study

**DOI:** 10.3390/biom15081074

**Published:** 2025-07-24

**Authors:** Francesca La Rosa, Simone Agostini, Elisabetta Bolognesi, Ivana Marventano, Roberta Mancuso, Franca Rosa Guerini, Ambra Hernis, Lorenzo Agostino Citterio, Federica Piancone, Pietro Davide Trimarchi, Jorge Navarro, Federica Rossetto, Arianna Amenta, Pierfausto Seneci, Silvia Sesana, Francesca Re, Mario Clerici, Marina Saresella

**Affiliations:** 1 IRCCS Fondazione Don Carlo Gnocchi, 20148 Milan, Italy; flarosa@dongnocchi.it (F.L.R.); ebolognesi@dongnocchi.it (E.B.); imarventano@dongnocchi.it (I.M.); rmancuso@dongnocchi.it (R.M.); fguerini@dongnocchi.it (F.R.G.); ahernis@dongnocchi.it (A.H.); lcitterio@dongnocchi.it (L.A.C.); fpiancone@dongnocchi.it (F.P.); ptrimarchi@dongnocchi.it (P.D.T.); jnavarro@dongnocchi.it (J.N.); frossetto@dongnocchi.it (F.R.); mario.clerici@unimi.it (M.C.); msaresella@dongnocchi.it (M.S.); 2 Chemistry Department, University of Milan, 20133 Milan, Italy; arianna.amenta@unmi.it (A.A.); pierfausto.seneci@unimi.it (P.S.); 3 School of Medicine and Surgery, University of Milano-Bicocca, 20900 Monza, Italy; mariasilvia.sesana@unimib.it (S.S.); francesca.re1@unimib.it (F.R.); 4 Department of Pathophysiology and Transplantation, University of Milan, 20122 Milan, Italy

**Keywords:** Alzheimer’s disease, inflammasome, NLRP3, glibenclamide, liposomes, rehabilitation

## Abstract

Background: Inflammasomes regulate the activation of caspases resulting in inflammation; inflammasome activation is dysregulated in Alzheimer’s disease (AD) and plays a role in the pathogenesis of this condition. Glibenclamide, an anti-inflammatory drug, could be an interesting way to down-modulate neuroinflammation. Methods: In this pilot study we verified with ex vivo experiments whether a glibenclamide-loaded nanovector (GNV) could reduce the NLRP3-inflammasome cascade in cells of AD patients. Monocytes isolated from healthy controls (HC) and AD patients were cultured in medium, alone or stimulated with LPS + nigericin in presence/absence of GNV. ASC-speck positive cells and inflammasome-related genes, proteins, and miRNAs expressions were measured. The polymorphisms of *ApoE* (Apolipoprotein E), specifically rs7412 and rs429358, as well as those of *NLRP3*, namely rs35829419, rs10733113, and rs4925663, were also investigated. Results: Results showed that ASC-speck+ cells and Caspase-1, IL-1β, and IL-18 production was significantly reduced (*p* < 0.005 in all cases) by GNV in LPS + nigericin-stimulated cells of both AD and HC. Notably, the *NLRP3* rs10733113 AG genotype was associated with excessive inflammasome-related gene and protein expression. GNV significantly down-regulates inflammasome activation in primary monocytes, at least at protein levels, and its efficacy seems to partially depend on the presence of the *NLRP3* rs10733113 genotype. Conclusions: All together, these results showed that GNV is able to dampen inflammation and NLRP-3 inflammasome activation in an ex vivo monocyte model, suggesting a possible role for GNV in controlling AD-associated neuroinflammation.

## 1. Introduction

Alzheimer’s disease (AD) is a neurodegenerative disorder marked by neuronal death, extracellular amyloid-beta (Aβ) plaques, and cognitive decline [1,2].

In AD, impaired clearance of amyloid β (Aβ) leads to its extracellular buildup and aggregation into amyloid plaques, causing neuronal toxicity [1], neuroinflammation, and damaged neurons [3,4,5]. Microglia, the brain’s resident macrophages, can degrade and phagocytose Aβ, but is variably described as dysfunctional due to amyloid overload [6], being non-essential for plaque development [7], or due to being limited in their ability to contain plaques and reduce toxicity in advanced AD [8,9]. The role of central nervous system (CNS) inflammation has been recognized since the initial identification of microgliosis [10,11]. Different studies have indicated that peripheral monocytes traffic into the inflamed brain parenchyma [12,13,14], a phenomenon that appears to be exacerbated in contexts such as post-ischemic injury [15] and neurodegenerative diseases like amyotrophic lateral sclerosis [16]. The role of microglia in Alzheimer’s disease is controversial [17], but several evidence seem to show that monocytes can cross the blood–brain barrier (BBB) [18,19] through interactions involving chemokines, adhesion molecules, and Aβ-RAGE signaling pathways, subsequently differentiating into macrophages within the brain [20,21]. Circulating monocytes account for approximately 6% of plaque-associated macrophages, predominantly entering the brain through entry points such as the choroid plexus, meninges, and perivascular spaces [22]. Experimental reduction of monocyte infiltration, for example via splenectomy, has been shown to increase amyloid-beta (Aβ) load, suggesting a role for monocytes in plaque clearance [23]. Conversely, monocytes can also secrete inflammatory cytokines that contribute to the persistent neuroinflammation observed in AD pathology. The microglial NLRP3 inflammasome responds to Aβ by producing IL-1β and IL-18, amplifying neuroinflammation; its deficiency promotes an anti-inflammatory M2 phenotype [24,25,26,27,28,29,30,31]. Overall, monocyte infiltration and neuroinflammation significantly impact AD progression, with peripheral monocytes aiding in Aβ clearance and potentially reducing pathology [1,2,3,4,5]. The NLRP3 inflammasome involves the adaptor protein ASC (coded by PYCARD gene), which assembles into “specks” for caspase-1 activation and cytokine production. NLRP3 gene and protein expression are modulated epigenetically, with miR-223-3p and miR-7-1-5p acting to suppress inflammasome activity [32,33,34]. The NLRP3 gene, located on chromosome 1q44, contains 9 exons and approximately 60 SNPs. Notably, the rs10733113 polymorphism has been linked to altered inflammasome activity in monocytes from AD patients, influencing inflammation, disease severity, and progression [35,36]. Therapeutic efforts have struggled mainly due to poor drug delivery across the BBB. Nanotechnology has enabled the development of liposome-based nanocarriers that can transport drugs to modulate neuroinflammation [37]. Engineered nanovectors (NVs), such as multifunctionalized liposomes carrying mApoE, can cross the BBB and release drugs locally in inflamed tissue, where matrix metalloproteinases (MMPs) are overexpressed. Among anti-inflammatory agents, glibenclamide inhibits NLRP3 inflammasome activation, reducing neuroinflammation and offering a potential new AD treatment [38,39]. This pilot study aimed to evaluate, in ex vivo, the effectiveness of glibenclamide-loaded nanovectors (GNV) in suppressing NLRP3 inflammasome activation in monocytes from AD patients and healthy controls.

## 2. Materials and Methods

### 2.1. Patients and Controls

Seventeen patients who fulfilled the inclusion criteria for the clinical diagnosis of AD were enrolled by the Rehabilitative Unit of Neurology clinic of IRCCS Fondazione Don Carlo Gnocchi, Milan, Italy. The clinical diagnosis of probable AD was performed according to the NINCDS-ADRDA work group criteria [40] and the DMS IV–R [41]. All subjects were characterized with neuropsychological tests, including the mini mental state examination (MMSE). All patients underwent a standard battery of examinations, a physical and neurological examination, screening laboratory blood tests, and neurocognitive evaluation. Exclusion criteria are as follows: presence of neurological or psychiatric conditions (e.g., Parkinson’s disease, multiple sclerosis, major depression, schizophrenia) or severe systemic diseases (e.g., heart failure, stroke, advanced liver or kidney diseases). Thirteen gender- and age-matched healthy controls (HC) were enrolled as well in the study. Inclusion criteria for HC are as follows: age between 70 and 85 years, no family history of dementia, and no evidence of acute or chronic neurologic and neuro-psychiatric diseases; no chronic inflammatory and autoimmune diseases; immunomodulatory drug free, absence of cognitive decline (MMSE score = 30). All HC were selected according to the SENIEUR protocol for immuno-gerontological studies of the European Community’s Control Action Programme on Aging [42]. The study was conducted in accordance with the Declaration of Helsinki and approved by the Ethics Committee of IRCSS Fondazione Don Carlo Gnocchi ((#9_25/07/2019, 25 July 2019).

### 2.2. Blood Sample Collection, DNA Extraction, and ApoE and NLRP3 Genotyping

Blood samples were obtained from all enrolled participants using vacutainer tubes containing ethylenediaminetetraacetic acid (EDTA) (Becton Dickinson & Co., Rutherford, NJ, USA). DNA was extracted by standard phenol/chloroform procedures, and stored at −20 °C until use. The DNA concentration of each sample was calculated by measuring the optical density at 260 nm wavelengths using a spectrophotometer (Nanovue, GE Healthcare, Chicago, IL, USA). The *Apolipoprotein E* (*ApoE*) gene encodes for three major ApoE protein isoforms, ɛ2, ɛ3, and ɛ4. The ɛ4 allele significantly increases Alzheimer’s disease (AD) risk and is linked to an earlier onset, while the ɛ2 allele offers a protective effect, reducing risk and delaying onset [43]. These isoforms are determined by specific allelic combinations of two single nucleotide polymorphisms (SNPs): rs7412 (T/C) and rs429358 (C/T): ε2 corresponds to the genotype rs429358 (T)/rs7412 (T), ε3 corresponds to rs429358 (T)/rs7412 (C), and ε4 corresponds to rs429358 (C)/rs7412 (C) [44].

Genotyping was performed by allelic discrimination real-time PCR by the pre-designed TaqMan™ probes (Thermo Fisher Scientific, Waltham, MA, USA), C____904973 (rs7412) and C___3084793 (rs429358) to determine *ApoE* genotypes, using the following PCR protocol: hot start at 95 °C for 10 min followed by 40 cycles consisting of denaturation at 94 °C for 15 s and annealing/extension at 60 °C for 1 min. Fluorescence detection was carried out at 60 °C. Genotyping assays were performed in a final reaction volume of 10 μL, containing 1 μL of genomic DNA (10 ng/μL) and TaqMan Genotyping Master Mix. Reactions were run on 96-well plates (CFX96 Real-Time PCR Detection System, Bio-Rad, Hercules, CA, USA). Each assay included positive controls representing all possible genotypes as well as a no-template (negative) control. The *NLRP3* (Nod Like Receptor Family Pyrin Domain Containing 3) gene encodes for a complex multimeric protein involved in the NLRP3 inflammasome. The gene contains several SNPs, and among them, the rs35829419, located in exon 3 (Q705K), and the rs10733113 and rs4925663, located in a potential regulatory region downstream of *NLRP3* [45], were chosen as commonly inflammasome-related polymorphisms. Also, these SNPs were genotyped by allelic discrimination real-time PCR, using the C__25648615_10 (rs35829419), the C__30713847_10 (rs10733113), and the C__26052022_10 (rs4925663) pre-designed TaqMan™ probes.

### 2.3. Peripheral Blood Mononuclear Cell Processing

Peripheral blood mononuclear cells (PBMCs) were isolated using a lymphocyte separation medium (Cedarlane, Hornby, Ontario, CA, USA) and washed twice with PBS at 1500 g for 10 min; the number of viable leukocytes was assessed using a TC20 Automated Cell Counter (Bio-Rad, Hercules, CA, USA). PBMCs were plated at a concentration of 2 × 10^6^/mL on tissue culture plates (6-well plates), and cultured in RPMI 1640 supplemented with 10% human serum, 2 mM L-glutamine, and 1% penicillin (Invitrogen, Ltd., Paisley, UK) overnight at 37 °C with 5% CO_2_ in a humidified environment to facilitate monocyte adhesion to the surface. The following day, the RPMI was changed and adherent monocytes were unstimulated (MED) or primed for 2 h with LPS (1 μg/mL) and followed by nigericin (5 µM) (Sigma-Aldrich, St. Louis, MO, USA) for 22 h in absence or presence of glibenclamide-loaded NV carrying mApoE and MMP-Sensitive Lipopeptides (GNV) (10 µM) that correspond to a concentration of 2.5 µM of glibenclamide (dissolved in RPMI). The concentrations of LPS, nigericin, and glibenclamide were chosen on the basis of our previous experiments [38,39]. Each experiment was also performed using glibenclamide alone as internal control. Nigericin is a microbial toxin derived from *Streptomyces hygroscopicus* and acts as a potassium ionophore; the release of inflammatory cytokines in response to nigericin has been demonstrated to be NLRP3 inflammasome-dependent [46,47].

At the end of incubation, supernatants were collected, and adherent monocyte cells were harvested by Accutase (CliniSciences, Nanterre, France) and their viability was determined for each experiment (pre- and post-stimulation) using a TC20 Automated Cell Counter (Bio-Rad, Hercules, CA, USA), by Tripan blue methodology.

### 2.4. Preparation and Characterization of GNVs

GNVs were prepared as described in [37,48]. In particular, 5 µmol of lipid mixture was dissolved in 9 mL of chloroform and was mixed with 0.5 mg of glibenclamide that was previously dissolved in methanol (0.13 mg/mL). Liposomes were prepared and purified as previously described [37,48]. Lipid recovery was assessed using the Stewart assay [49]. Briefly, lipids were extracted, dissolved in chloroform, and mixed with ammonium ferrothiocyanate, forming a colored complex with phospholipids. This complex remained in the organic phase, which was then collected and measured at 485 nm. The phospholipid content was quantified by comparing absorbance values to a standard curve. The encapsulation efficiency (EE%) was calculated as previously described [50].

### 2.5. Intracellular ASC Protein Staining and Imaging Flow Cytometry by FlowSight AMNIS

PBMCs (2 × 10^6^), stimulated as previously described, were incubated with 5 μL (25 μg/mL) of FITC-conjugated anti-human NLRP-3 (clone, mouse isotype) monoclonal antibody for 60 min at 4 °C. Afterward, cells were washed with PBS, centrifuged, and then permeabilized with 100 μL of Saponin in PBS (0.1%) (Life Science VWR, Lutterworth, Leicestershire, UK). Subsequently, 5 μL (25 μg/mL) of PE-conjugated anti-human ASC (clone HASC-71, mouse IgG1 isotype, Biolegend, San Diego, CA, USA) monoclonal antibody was added to the samples and incubated for 60 min at 4 °C. Cells were then rinsed with PBS, centrifuged at 1500 g for 10 min at 4 °C, fixed with 100 μL of 1% PFA in PBS (BDH, UK) for 15 min, washed again with PBS, centrifuged at 1500 rpm for 10 min at 4 °C, resuspended in 50 μL of cold PBS, and analyzed via AMNIS FlowSight. The instrument acquires 2000 events per sample and performs the analysis using the IDEAS analysis software version 6.2 (Amnis Corporation, Seattle, WA, USA). The formation of the apoptosis-associated speck-like protein containing CARD (ASC) specks was evaluated through the internalization feature, employing a mask that encompasses the entire cell defined by the bright field (BF) image, as previously described [51].

### 2.6. Cytokines Production and Caspase-1(p20) Release

Simple Plex assays for the quantification of IL-18 (catalog: SPCKB-PS-000501), caspase-1 (p20 subunit) (catalog: SPCKB-PS-003613), and IL-1β (catalog: SPCKB-PS-000216) were performed using an automated immunoassay platform (ELLA) (Biotechne, Minneapolis, MN, USA), which utilizes a microfluidic cartridge for the automatization of all immunoassay steps. Collected supernatants from monocyte cells were centrifuged to eliminate debris and immediately analyzed following the manufacturer’s instructions. The detection limit was: 0.064 pg/mL for human IL-1β; 0.2 pg/mL for IL-18; and 0.04 pg/mL for caspase-1. Optical density (OD) was measured at 450/620 nm. All experiments were conducted in duplicate.

### 2.7. RNA Extraction, and Analysis of mRNA and miRNA Expression by ddPCR

From the monocyte cells treated as described in paragraph 4.4, total RNA was extracted by a commercial kit (miRNeasy cell/tissue Advanced Micro kit, Qiagen, Hilden, Germany), by semi-automated robot-work station Qiacube (Qiagen, Hilden, Germany). mRNA and miRNA concentrations were measured by a fluorometer (Qubit 2.0, ThermoFisher, Foster City, CA, USA) using specific kits (for mRNA: RNA HS assays, High Sensitivity, ThermoFisher; for miRNA: Qubit^®^ miRNA assay, Thermofisher). miR-223-3p and miR-7-1-5p concentration, and NLRP3, IL-1β, PYCARD (i.e., the gene that codes ASC protein), caspase-1, IL-18 gene expression, were quantified by droplet digital PCR (ddPCR QX200, Bio-Rad). For miRNAs quantification, 4 µL of extracted RNA was converted in cDNA with the miRCURY LNA RT Kit (Qiagen), according to the manufacturer’s protocol. After this, miRNA LNA™ PCR-specific primer sets were mixed with appropriate diluted (1:60) cDNA (3 µL for miR-7-1-5p, assay number: YP00205877; 1.5 µL for miR-223-3p, assay number: YP00205986) and with ddPCR EvaGreen Supermix (Bio-Rad). For gene expression quantification, 5 μL of extracted RNA (1:100) were used for a one-step RT-ddPCR, mixed with dithiothreitol (DDT, 15 nM), RT-ddPCR Supermix (1x), reverse transcripate enzyme (1x), specific primers (900 nM), and FAM- or HEX-fluorescent probes (250 nM probe). In particular, *NLRP3* (dHsaCPE5058640, FAM-fluorescent probe) and *IL-1β* (dHsaCPE5057883, HEX-fluorescent probe) were detected in the same reaction, as well as *caspase-1* (dHsaCPE5031522, FAM-fluorescent probe) and *IL-18* (dHsaCPE5038347, HEX-fluorescent probe); *PYCARD* (dHsaCPE5033926, FAM-fluorescent probe) was detected alone. The ddPCR workflow, conditions, and data analyses for gene and miRNAs were previously described [40]. The ddPCR results were analyzed by QuantaSoft software (Bio-Rad, version 1.7.4.0917).

### 2.8. Statistical Analysis

Data distribution was tested by Shapiro–Wilk test. Normally distributed data were expressed as mean ± standard deviation, and comparisons among groups were analyzed by Student *t*-test. Not normally distributed data were expressed as median and interquartile range (IQR: 25th and 75th percentile), and comparisons between the different culture conditions were made using a 2-tailed Mann–Whitney U test. The Hardy–Weinberg equilibrium (HWE) for the *ApoE* and *NLRP3* SNPs distribution was calculated using a Chi-square method in both AD patients and controls. Allele and genotype distributions between AD patients and HC were compared using 2 × N contingency tables. The association between SNPs and AD/HC was evaluated using the odds ratio (OR) and its 95% confidence interval (CI). *p*-values were corrected using the Bonferroni method for *n–1* degrees of freedom (df) (reported as *pc*). When the expected frequency in any cell was less than 5, Fisher’s exact test was applied, and the corresponding *p*-value was reported as *pf*. The genotype association in AD patients and HC with cytokine and miRNA levels was tested with the parametric ANOVA. *p*-values were considered significant when ≤ 0.05. The statistical analyses were accomplished using commercial software (MedCalc Statistical Software, version 14.10.2, Ostend, Belgium, GraphPad version 10.3.1 Software Inc., San Diego, CA, USA, SPSS, version 28.0, IBM Corp. Armonk, NY, USA) and the openEpi https://www.openepi.com (accessed on 18 June 2025).

## 3. Results

### 3.1. Clinical Characteristics of AD and HC Subjects Included in the Study

Demographic, clinical, and genetic characteristics of the AD patients and healthy control (HC) enrolled in the study are presented in Table 1. No differences were observed in gender, age, or years of education between AD and HC, whereas global cognitive levels (MMSE) were significantly reduced in AD patients (median 20.0 ± 3.0) compared to healthy controls (30.0) (*p* < 0.0001), as per the inclusion criteria. No statistical differences were found between patients and HC *ApoE4* carriers (17.6% and 11.5%, respectively).

### 3.2. ApoE and NLRP3 Genotypic Characterization

In Table 2 the genotype and allelic distribution of *ApoE* and *NLRP3* genes were shown in both AD and HC subjects; the variables were in the Hardy–Weinberg equilibrium of each group. No statistically significant differences were observed between AD patients and healthy controls in terms of *ApoE* genotype distribution (pc = 0.32). Additionally, when subjects were categorized as *ApoE4* carriers and non-carriers, the proportion of *ApoE4+* individuals did not significantly differ between AD patients (35.3%) and HC (23.1%) (*p* = 0.51).

In addition, the distribution of *NLRP3* genotypes was also compared between the two groups. The rs10733113 AG polymorphism was found more frequently in AD patients (41.2%) than in HC (15.4%), but this difference did not reach statistical significance (pf = 0.2).

### 3.3. Characterization of GNV

The GNV characterization (Table 3) indicated a consistent size distribution (PolyDispersity Index, PDI ≤ 0.2) with a ≤ 160 nm diameter and a negative surface charge, implying that nanovectors are stable and not unlikely to aggregate. The encapuslation efficiency, EE%, of glibenclamide was 75 ± 9%. Stability of the GNV was assessed by tracking their size, PDI, and ζ-potential over time. The results demonstrated that there were no significant changes in size and PDI over a period of 10 days.

### 3.4. GNV-Effects on Inflammasome Gene and miRNAs Expression in LPS-Primed and Nigericin-Stimulated Monocytes

**Figure 1:**
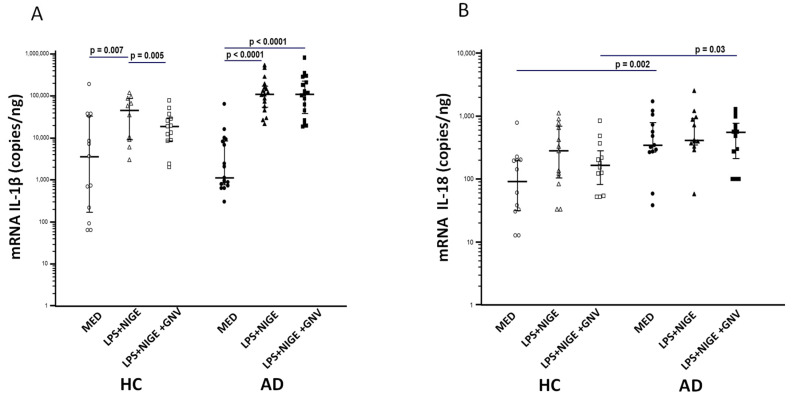
IL-1-β (**A**), and IL-18 (**B**) mRNA expression in unstimulated (MED) or LPS + nigericin (NIG)-stimulated monocytes isolated from Alzheimer’s disease patients (AD) or healthy controls (HC) in presence/absence of glibenclamide-loaded nanovector (GNV). Median value and interquartile range are represented.

**Figure 2:**
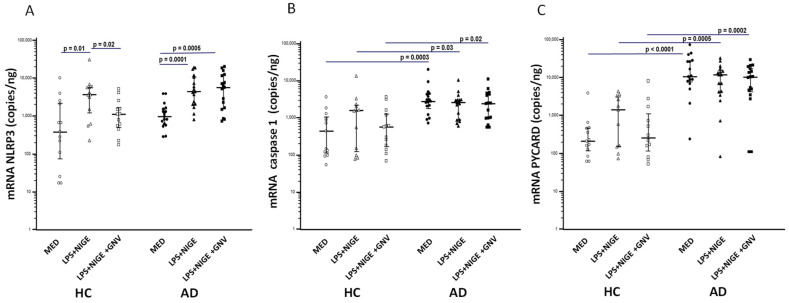
NLRP3 (**A**), Caspase-1 (**B**), and PYCARD (**C**) mRNA expression in unstimulated (MED) or LPS + nigericin (NIG)-stimulated monocytes isolated from Alzheimer’s disease patients (AD) or healthy controls (HC) in presence/absence of glibenclamide-loaded nanovector (GNV). Median value and interquartile range are represented.

mRNAs expression of inflammasome-related genes (*NLRP3*, *IL-1β*, *caspase-1*, *PYCARD*, *IL-18*) was measured in monocytes stimulated with the aforementioned conditions by ddPCR, and was compared to unstimulated cells. All the results are represented in Figure 1 and Figure 2. Unstimulated monocytes isolated from AD showed a significantly higher mRNA expression of *caspase-1*, *PYCARD*, and *IL-18* compared to that measured in HC (*p* ≤ 0.001 for all genes), confirming that an excessive activation of the NLRP3 inflammasome is present in AD. As expected, *NLRP3* and IL-1β expression was significantly increased in those LPS-primed and nigericin-stimulated compared to unstimulated monocytes of both AD and HC subjects (*p* < 0.05 for all the comparisons). GNVs addition significantly reduced *NLRP3* and *IL-1β* gene expression in HC monocytes (*NLRP3*: *p* = 0.02; *IL-1β*: *p* = 0.05 *vs*. LPS + nigericin-stimulated cells) but it did not have an effect in AD cells. *Caspase-1*, *IL-18*, and *PYCARD* gene expression were increased in HC monocytes after activation with LPS + nigericin and were down regulated by GNV, although these variations did not reach the statistical significance, probably due to the limited number of subjects; no major differences were observed in AD cells.

**Figure 3:**
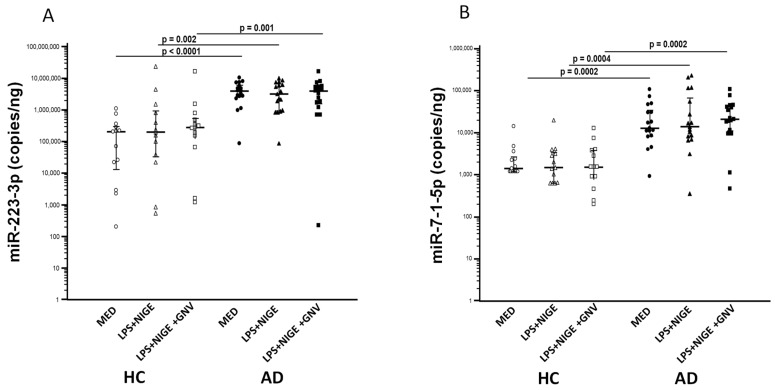
miR-223-3p (**A**) and miR-7-1-5p (**B**) expression in unstimulated (MED) or LPS+ nigericin (NIG)-stimulated monocytes isolated from Alzheimer’s disease subjects (AD) and healthy controls (HC) in presence/absence of glibenclamide-loaded nanovector (GNV). Median value and interquartile range are represented.

miR-223-3p and miR-7-1-5p expression were measured by ddPCR as well in both HC and AD cells. Expression of both miRNAs was significantly higher in AD vs. HC monocytes in all culture conditions (unstimulated, LPS + nigericin, and LPS + nigericin + GNV; *p* < 0.005 for all comparisons) (Figure 3A,B), without significant difference among other conditions. No associations were found with age or gender.

### 3.5. GNV-Effects on Inflammasome and Downstream Protein Activation in Monocytes

**Figure 4:**
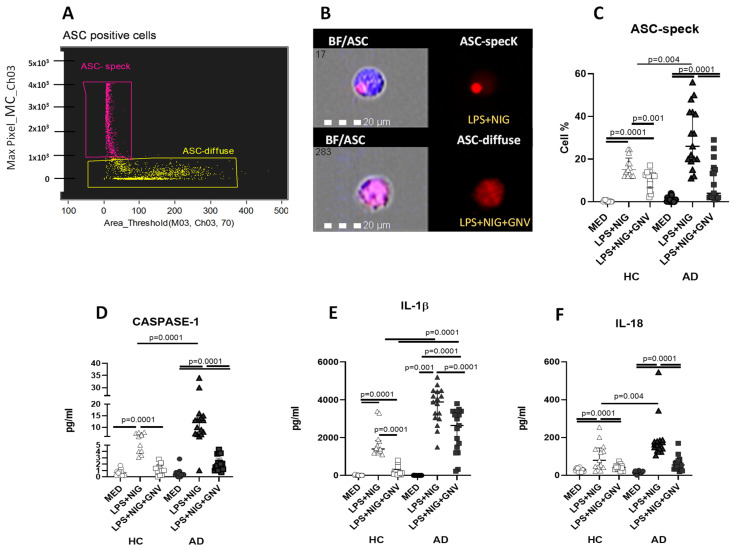
Representative images of ASC-speck formation collected by unstimulated (MED); LPS + nigericin (NIG)-stimulated cells in presence/absence of glibenclamide-loaded nanovector (GNV) of AD patients (**A**,**B**). (**A**) ASC positive cells including ASC-diffuse (yellow region) with max pixel score < 10^3^; (**B**) ASC-speck (pink region) with max pixel > 10^3^. ASC-speck formation was analysed by IDEA-AMNIS software version 6.2 that recognizes two different diameters of the spot area (AREA-Threshold) of ASC fluorescence inside cells: diffuse or spot (speck) that can separate all ASC-positive cell population in ASC-speck spot cells or ASC-diffuse cells. In ASC-speck cell, the spot showed a small area and high max pixel; conversely in ASC-diffuse cell, the fluorescence showed a large area and low max pixel. The first column shows cells in brightfield (BF) merged with ASC; second column shows ASC-PE fluorescence. Monocyte percentage expressing ASC-speck formation (**C**) and caspase-1 (**D**); IL-1β (**E**) and IL-18 (**F**) production in supernatants of unstimulated (MED) or LPS + nigericin (NIG)-stimulated cells in presence/absence of glibenclamide-loaded nanovector (GNV) of Alzheimer’s disease patients (AD) and healthy control (HC). Data are expressed as median and interquartile range are represented.

ASC-specks formation identifies a functional inflammasome complex assembly that creates a multitude of potential caspase-1 activation sites, thus serving as a signal amplification mechanism for inflammasome-mediated cytokine production. The effect of GNV on ASC-speck formation was investigated in LPS-primed and nigericin-stimulated monocytes of AD and HC individuals by imaging flow cytometry. Results showed that GNV addition significantly reduced the percentage of ASC-speck positive cells of both AD and HC donors when LPS + nigericin-stimulated and unstimulated conditions were compared (*p* < 0.005 in both cases) (Figure 4A–C). Caspase-1 (p20), IL-1β, and IL-18 concentration was quantified next in supernatants by ELLA. Results showed that GNV significantly reduced in LPS-primed and nigericin-stimulated cells: (1) caspase-1 (p20) (AD and HC *p* < 0.0001) (Figure 4D); (2) IL-1 β (AD *p* < 0.005; HC *p* < 0.0001) (Figure 4E); and (3) IL-18 (AD *p* < 0.0001; HC *p* = 0.003) (Figure 4F) production.

These parameters were subsequently analyzed by comparing AD and HC cells. There was a similar down-modulation of GNV on ASC formation, and the release of activated caspase 1 (p20) and IL-18 production was observed in both groups. Conversely, IL-1β production was not reduced by GNV in AD compared to HC cells (*p* < 0.0001) (Figure 4A–F). No associations were found with age or gender.

### 3.6. Association Among NLRP3 Polymorphisms, miRNAs and Gene Expression, and Protein Concentrations

**Figure 5:**
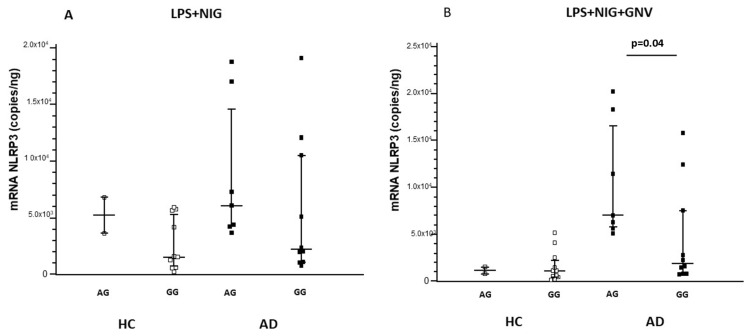
NLRP3 gene expression in monocytes of Alzheimer’s disease patients (AD) and healthy controls (HC) after LPS primer and nigericin stimulation (LPS + NIG) (**A**) and after GNV addiction (GNV) (**B**), divided for *NLRP3* rs10733113 genotype. Median value and interquartile range are represented.

**Figure 6:**
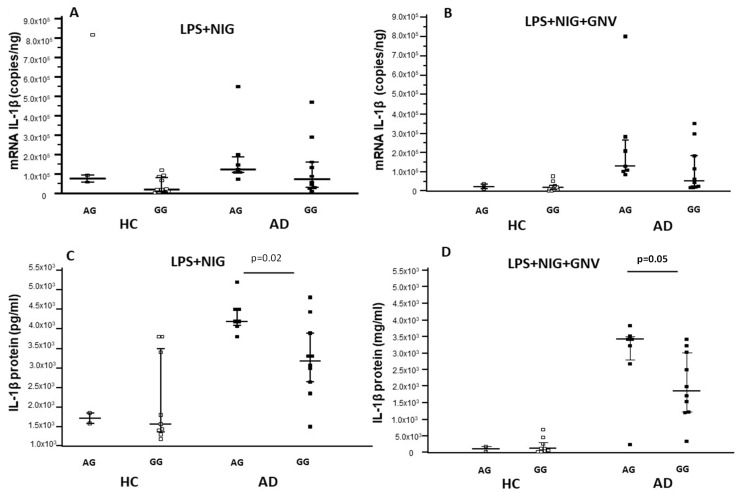
IL-1β gene expression and protein concentration in monocytes of Alzheimer’s disease patients (AD) and healthy controls (HC) after LPS primer and nigericin stimulation (LPS + NIG) (**A**,**C**) and after GNV addiction (GNV) (**B**,**D**), divided for NLRP3 rs10733113 genotype. Median value and interquartile range are represented.

**Figure 7:**
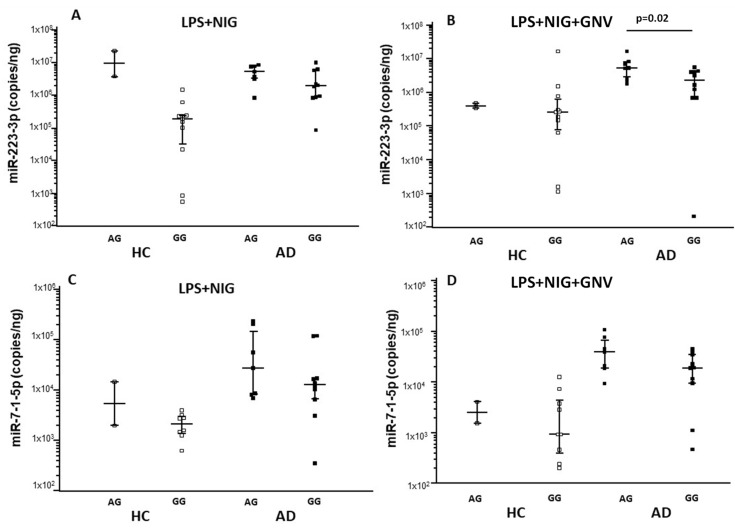
miR-223-3p and miR-7-1-5p concentration in monocytes of Alzheimer’s disease patients (AD) and healthy controls (HC) after LPS primer and nigericin stimulation (LPS + NIG) (**A**,**C**) and after GNV addiction (GNV) (**B**,**D**), divided for NLRP3 rs10733113 genotype. Median value and interquartile range are represented.

The results above showed that NLRP3 mRNA expression was significantly increased by LPS + Nig stimulation in AD compared to HC monocytes; however, GNV reduced NLRP3 mRNA copies in HC but not in AD cells. Analyzing the relation between *NLRP3* single nucleotide polymorphisms (SNPs) and gene expression in monocytes isolated from all the enrolled subjects, we found that NLRP3 mRNA was significantly more expressed in every culture condition by cells carrying the rs10733113 AG compared to the GG genotype (*p* = 0.01 for all comparisons). Moreover, a significantly higher expression of IL1-β mRNA and protein was observed in all culture conditions in cells of individuals carrying the rs10733113 AG compared to the GG genotype (*p* < 0.05 for all comparisons) (Supplementary Materials Table S1 and Figure S1). Similar results were found for miRNAs expression: miR-223-3p and miR-7-1-5p were significantly more expressed in all culture conditions by monocytes of subjects carrying the rs10733113 AG compared to the GG genotype (*p* < 0.05 for all comparisons) (Supplementary Materials, Table S1, Figure S1). To evaluate the possible impact of these results on AD pathogenesis, AD and HC data were analyzed separately. Results were confirmed in AD patients alone. In patients, but not in HC, NLRP3 mRNA, miR-223-3p, and IL1β were significantly up-regulated in individuals carrying the rs10733113 AG compared to GG genotype (*p* = 0.04, *p* = 0.02, and *p* = 0.05, respectively) (Figure 5, Figure 6 and Figure 7). These results are summarized as Supplementary Materials (Table S1 and Figure S1). No associations were found with age or gender.

## 4. Discussion

In AD, circulating monocytes with inflammatory phenotypes can cross the BBB and differentiate into microglia in the CNS, possibly in the attempt to remove amyloid-beta (Aβ) from senile plaques [18,52,53,54]. These activated monocytes produce inflammatory cytokines such as IL-18 and IL-1β, which are the final products of inflammasome complex activation, contributing to AD-associated neuroinflammation. We have previously found that AD-peripheral monocytes are characterized by an inflammatory profile and that these cells express binary complexes formed by Aβ peptides and MHC molecules, possibly initiating an Aβ-specific acquired immune response [55]. Finally, we showed that the NLRP3 and NLRP1 inflammasomes are indeed activated in the peripheral monocytes of AD patients [56]. These findings prompted us to analyze possible NLRP3 inflammasome inhibitors. By taking advantage of the complex signaling cascade of the NLRP3 inflammasome, several targets can be used for its inhibition. We have previously shown that nucleoside reverse transcriptase inhibitors (NRTI) reduce NLRP3 assembly in PBMCs of AD patients and enhance amyloid-β autophagy in macrophages, potentially offering therapeutic benefits for Alzheimer’s disease [57]. miR-223-3p can hamper NLRP3 activation as well; This miRNA targets a binding site in the 3′-UTR of NLRP3 mRNA [32]. Biomedical engineering has substantially contributed to our understanding of the physiological barriers to efficient drug delivery and has contributed to the development of new modes of molecule delivery that have entered clinical practice. Drug delivery systems also include how molecules are ‘packaged’—e.g., micelle or nanoparticle—to protect them from degradation and allowing their travel throughout in the body. We verified the efficacy of glibenclamide-loaded nanovectors (GNV) to dampen NLRP3 inflammasome activation. Consistently, with previous data obtained on an in vitro THP-1 cell model [38], present results show that GNVs induce a consistent transcriptional down-regulation of inflammasome-related genes in HC monocytes, but not in AD monocytes. In contrast with these results, GNVs only marginally affected the concentration of two miRNAs (miR-223-3p and miR-7-1-5p) targeting NLRP3. This was unexpected and might be explained by the relatively small number of subjects enrolled in our study. To note, in an in vitro study we have previously observed that NRTI modulates NLRP3-related genes differently (*PYCARD*, *caspase-1*, *IL-18* and *IL-1β*), and miR-223-3p and miR-7-1-5p expression in monocytes of AD and HC individuals, measured by ddPCR [58], suggest that the regulatory mechanisms are different in relation of the disease state. Importantly, the present study confirms an altered regulatory mechanism in monocytes of AD subjects, where miR-223-3p and miR-7-1-5p were consistently up-regulated, probably in an attempt to dampen the strong inflammasome activation. Next, to study the GNV effect on protein release, we measured ASC-speck formation and the cytokines produced by downstream NLRP3 complex activation. Results indicated that GNV causes a significant decrease in (1) ASC-speck cell percentage, (2) activated caspase-1, and (3) IL-1β and IL-18 production in HC and AD monocytes, confirming previous results [39]. When data were analysed in AD and HC separately, results were as follows: (1) a significantly higher inflammasome activation in AD compared to HC monocytes; and (2) a similar dampening effect on NLRP3 activation in both AD and HC as stated by the reduction of both ASC-speck formation and caspase-1 and IL-18 production. The ASC speck facilitates proximity-induced autocatalytic activation of caspase-1. Active caspase-1 processes pro-IL-1β and pro-IL-18 into their mature forms, leading to cytokine secretion. The speck may also play a role in amplifying the inflammatory response and can be a target for therapeutic modulation.

Notably, IL-1β production was significantly decreased by GNV in HC but not in AD, probably because the complex and highly redundant IL-1β production pathways are overactivated in AD [59,60,61,62]. To note, our study showed that in AD monocytes the mRNA expression and protein production of NLRP3-related genes were not directly and linearly associated, as proteins were significantly down-regulated, but not mRNA. It is possible to hypothesize that this inconsistency is due to the intervention of other post-translational modifications, such as ubiquitination and phosphorylation [10,14] or autophagy-related pathway activation as previously investigated [41,59], but future studies are fundamental to better explain this point. A specific *NLRP3* rs10733113 AG genotype result was significantly associated with increased IL-1β gene and protein expression, as well as with higher *NLRP3* gene expression. Notably, in levels of miR-223-3p and miR-7-1-5p, two miRNAs known to upregulate *NLRP3* activation [38] were greatly increased in individuals carrying the *NLRP3* rs10733113 AG. This *NLRP3* rs10733113 SNP is located in a potential regulatory region downstream of the *NLRP3* gene, and it is a commonly inflammasome-related polymorphism [45]. These findings are in line with recent studies showing a significant association of the minor allele rs10733113 (A) with higher *NLRP3* expression and IL-1β production [63]. Because the rs10733113 AG genotype was more frequently observed in AD it is tempting to speculate that the different effects of GNV on IL-1β production in AD and HC is at least partially due to a different genetic background.

The higher frequency of the rs10733113 AG genotype seen in AD and the inflammatory status typical of the disease, together with post-transcriptional modification, might also explain why GNV robustly reduced the expression of NLRP3 proteins but only marginally affected that of *NLRP3* mRNAs.

We cannot exclude the possibility that the observed association between the *NLRP3* rs10733113 GA genotype and miRNA or protein concentrations may be influenced by other risk factors, such as *ApoE4* or additional genes interacting with *NLRP3*. Although the *ApoE* genotype was assessed in our study, the current sample size did not allow for stratified analyses between *ApoE4* carriers and non-carriers within genotype or molecular expression groups. Future studies involving larger and adequately powered cohorts will be necessary to investigate the potential interactive effects between *ApoE4* and *NLRP3* polymorphisms on miRNA and protein expression levels in AD patients.

To note, as glibenclamide is used for the treatment of type 2 diabetes, and one of the side effects of its use is hypoglycemia; future studies are needed to verify whether treatment with GNVs leads to any metabolic changes in AD patients.

## 5. Conclusions

To summarize, this pilot study shows that GNV is able to dampen the inflammation and activation of NLRP-3 inflammasome-related proteins in an ex vivo monocyte model. Moreover, the rs10733113 variant in monocytes from AD patients, carrying the A allele of rs10733113, might exhibit exaggerated NLRP3 activation, contributing to chronic inflammation and neurodegeneration. These results clearly have some limitations, including the small sample size, the lack of data on biochemical alterations in AD patients, and the fact that results are based on data obtained in only one neuroinflammatory condition and only one cell type. These data, nevertheless, are an initial exploration of possible new therapeutic approaches to neurodegenerative disease-associated neuroinflammation using drug-delivery nanovectors.

## Figures and Tables

**Table 1:** Demographic, clinical, and genetic characteristics of the individuals enrolled in the study.

	AD Patients	HC
**N**	17	13
**Gender (M:F)**	6:11	4:9
**Age (years)**	79.3 ± 2.5	76.4 ± 3.0
**MMSE** **Level of education (years)**	20 ± 3 9.1 ± 4.6	30 10.0 ± 5.6
**Amyloid-β in CSF (pg/mL)**	390 ± 132	-
**Total-τ in CSF (pg/mL)**	607 ± 270	-
**Phospho-τ in CSF (pg/mL)**	104 ± 40	-
***ApoE*** **4-carriers (%)**	17.6	11.5

Data are expressed as mean ± standard deviation. AD: Alzheimer’s Disease; HC: Healthy Controls; M: male; F: female; CSF: Cerebrospinal fluid; MMSE: Mini-Mental State Examination; *ApoE: Apolipoprotein E*.

**Table 2:** *ApoE* and *NLRP3* SNPs.

		AD Patients		HC	
*ApoE*	Genotypes	N = 17	%	N = 13	%
	ε2/ε3	0	0.0	2	15.4
	ε2/ε4	1	5.9	0	0.0
	ε3/ε3	11	64.7	8	61.5
	ε3/ε4	5	29.4	3	23.1
					pc = 0.6 2 df
	ε4+	6	35.3	3	23.1
	ε4−	11	64.7	10	76.9
					pf = 0.5 1
***NLRP3***	rs35829419				
	C C	17	100	12	92.3
	C A	0	0.0	1	7.7
	A A	0	0.0	0	0.0
					pf = 0.4
	rs10733113				
	G G	10	58.8	11	84.6
	G A	7	41.2	2	15.4
	A A	0	0.0	0	0.0
					pf = 0.2
	rs4925663				
	C C	9	52.9	4	30.8
	C T	6	35.3	6	46.1
	T T	2	11.8	3	23.1
					pc = 0.4 2 df

Data are expressed as absolute numbers and percentages. AD: Alzheimer’s Disease; HC: Healthy Controls; ApoE: Apolipoprotein E; NLRP3: NLR family pyrin domain containing 3. pc: *p*-value corrected for degrees of freedom (df) using the Bonferroni method; pf: *p*-value from Fisher’s exact test. Fisher’s exact test was applied when the expected frequency in any cell was less than 5 (in 2 × 2 contingency table).

**Table 3:** Size distribution, diameter, and ζ-potential of the multifunctionalized GNV formulations.

	Diameter (nm)	PDI	ζ-Potential (mV)	EE (%)
**GNV**	145.1 ± 2.6	0.19	−29.5 ± 2.3	
	152.5 ± 2.5	0.20	−31.2 ± 3.4	75 ± 9

Data are expressed as mean ± standard deviation. EE: Encapsulation Efficiency; mV: milliVolt; nm: nanometers; PDI: PolyDispersity Index.

## Data Availability

The data presented in this study are available on request from the corresponding author. The data are not publicly available due to ethical restrictions.

## Supplementary Materials

The following supporting information can be downloaded at: https://www.mdpi.com/article/10.3390/biom15081074/s1, Figure S1: *NLRP3* (A) and *IL-1β* (B) gene expression, *IL-1β* protein concentration (C), miR223-3p (D), and miR-7-1-5p (E) expression in monocytes of enrolled subjects after LPS primer and Nigericine stimulation (LPS+NIG) and after GNV addiction (GNV) divided for NLRP3 rs10733113 genotype (AG, black dots; GG, white dots); Table S1: miR-7-1-5p and miR-223-3p expression, *NLRP3* and *IL-1β* gene expression, and IL-1β concentration in monocyte cells collected from Alzheimer’s diseases subjects (AD) and healthy controls (HC), and with or without treatment, splitted on the base of NLRP3 rs10733113 polymorphism.
