# Supplementary material for: Engineered Glibenclamide-Loaded Nanovectors Hamper Inflammasome Activation in an Ex Vivo Alzheimer’s Disease Model—A Novel Potential Therapy for Neuroinflammation: A Pilot Study
Source: Biomolecules. 2025 Jul 24;15(8):1074. doi: 10.3390/biom15081074 (PMC12383602; doi:10.3390/biom15081074)

Glibenclamide-Loaded Engineered Nanovectors as New Therapeutic Strategy in modulating inflammasome-mediated inflammatory processes: in Alzheimer's Disease

Francesca La Rosa[1], Simone Agostini[1*], Elisabetta Bolognesi[1], Ivana Marventano[1], Roberta Mancuso[1], Franca Rosa Guerini[1], Ambra Hernis[1], Lorenzo Agostino Citterio[1], Milena Zanzottera[1], Federica Piancone[1], Cristina Agliardi[1], Pietro Trimarchi[1], Jorge Navarro[1], Federica Rossetto[1], Arianna Amenta[2], Pierfausto Seneci[2], Francesca Re[3], Mario Clerici[1,4] and Marina Saresella[1]

[1]IRCCS Fondazione Don Carlo Gnocchi, Milan, Italy;

[2]Chemistry Department, University of Milan, Italy;

[3]School of Medicine and Surgery, University of Milan-Bicocca, Italy;

[4]Pathophysiology and Transplantation, University of Milan, Milan, Italy

#Present address: University of Pavia, PhD National Programme in One Health approaches to infectious diseases and life science research, Department of Public Health, Experimental and Forensic Medicine, Pavia, Italy, and University of Milan, Department of Biomedical and Clinical Sciences, Milan, Italy

*corresponding Author

Supplementary Materials

Table S1. miR-7-1-5p and miR-223-3p expression, NLRP3 and IL-1 β gene expression, and IL-1β concentration in monocyte cells collected from Alzheimer's diseases subjects (AD) and healthy controls (HC), and with or without treatment, splitted on the base of NLRP3 rs10733113 polymorphism. Data are reported as median and interquartile (IQR). AD: Alzheimer's Disease patients; HC: healthy controls; LPS: lipopolysaccharide; NIG: nigericin; GNV: Glibenclamide-loaded Nanovectors.

			AD			HC			TOTAL	
NLRP3 rs10733113		AG (7)	GG (10)	p-value	AG (2)	GG (11)	p-value	AG (9)	GG (21)	p-value
Variable	conditions	Median, IQR	Median, IQR		Median, IQR	Median, IQR		Median, IQR	Median, IQR	
**NLRP3 mRNA (copies/ng)**	unstimulated	800, 720-1280	1200, 551-1540	0.81	1024, 0-/	380, 108-2540	0.64	800, 504-1664	656, 279-1880	0.76
	LPS+NIG	6080, 4280-14600	2240, 1120-10480	0.16	5230, 3660-6800	1520, 787-5300	0.23	6080, 4100-9720	2000, 1103-5700	**0.01**
	LPS+NIG+GNV	7040, 5780-16600	1920, 840-7520	**0.04**	1168, 796-1540	1098, 465-2246	0.77	6320, 4225-13160	1480, 680-3120	**0.01**
**IL-1β mRNA (copies/ng)**		AG (7)	GG (10)	p-value	AG (2)	GG (11)	p-value	AG (9)	GG (21)	p-value
	unstimulated	2080, 800-1000	1040, 640-7200	0.47	18777, 0-/	3650, 217-32600	0.77	2080, 760-12920	1120, 640-8330	0.69
	LPS+NIG	122400, 108400-186400	73200, 30080-162400	0.23	76700, 59000-94400	21857, 9530-82300	0.41	109600, 89400-160800	46160, 18042-101200	**0.02**
	LPS+NIG+GNV	128000, 104200-261800	53000, 22320-182400	0.13	24239, 12280-36200	19021, 8433-27505	0.77	109600, 72650-225400	25200, 16977-65500	**0.02**
**IL-1β protein (pg/ml)**		AG (7)	GG (10)	p-value	AG (2)	GG (11)	p-value	AG (9)	GG (21)	p-value
	unstimulated	0.5 0.0-1.0	0.5 0.0-10.0	0.89	3.5, 1-/	12.0, 5.0-24.0	0.22	0.6, 0.0-3.5	8.2, 0.0-14.0	0.19
	LPS+NIG	4200, 4095-4500	3180, 2645-3890	**0.02**	1716, 1583-1850	1450, 1375-3500	0.91	4200, 3312-4500	3000, 1515-3700	**0.02**
	LPS+NIG+GNV	3400, 2784-3475	1830, 1198-2995	0.05	102, 24-180	126, 54-309	0.58	3200, 216-6425	696, 127-1896	0.08
**miR-7-1-5p (copies/ng)**		AG (7)	GG (10)	p-value	AG (2)	GG (11)	p-value	AG (9)	GG (21)	p-value
	unstimulated	30400, 11120-49280	12000, 7020-25460	0.19	2360, 0-/	1400, 0-23120	1	11360, 4600-40080	3600, 1183-12800	**0.04**
	LPS+NIG	27920, 8420-168060	12920, 6640-17520	0.36	8400, 2000-14800	2214, 1381-3063	0.29	14800, 8000-92980	3650, 1628-13840	**0.04**
	LPS+NIG+GNV	39440, 18940-68820	18920, 9440-35040	0.19	2812, 1544-4080	919, 405-4665	0.48	20800, 8040-52540	3760, 919-19060	**0.04**
**miR-223-3p (copies/ng)**		AG (7)	GG (10)	p-value	AG (2)	GG (11)	p-value	AG (9)	GG (21)	p-value
	unstimulated	4848000, 2189200-7264000	3300800, 1078400-5400000	0.32	546500, 200-/	209000, 47450-317971	0.92	3131200, 1902400-6464000	738400, 204400-3920000	0.07
	LPS+NIG	5424000, 3367600-7776000	1991200, 902400-5920000	0.16	13512000, 3824000-23200000	196667, 42338-238823	0.23	5424000, 3635600-8040000	60800, 142300-1942800	**0.001**
	LPS+NIGE+GNV	5392000, 3110800-7896000	2467200, 697600-4144000	**0.02**	411640, 344681-478600	262857, 86835-660724	0.41	5248000, 149125-7368000	697600, 181335-3421600	**0.02**

Figure S1. NLRP3 (A) and IL-1β (B) gene expression, IL-1 β protein concentration (C), miR223-3p (D), and miR-7-1-5p (E) expression in monocytes of enrolled subjects after LPS primer and Nigericine stimulation (LPS+NIG) and after GNV addiction (GNV) divided for NLRP3 rs10733113 genotype (AG, black dots; GG, white dots). Median value and interquartile range are represented. Statistical significance is shown: *=p<0.05.

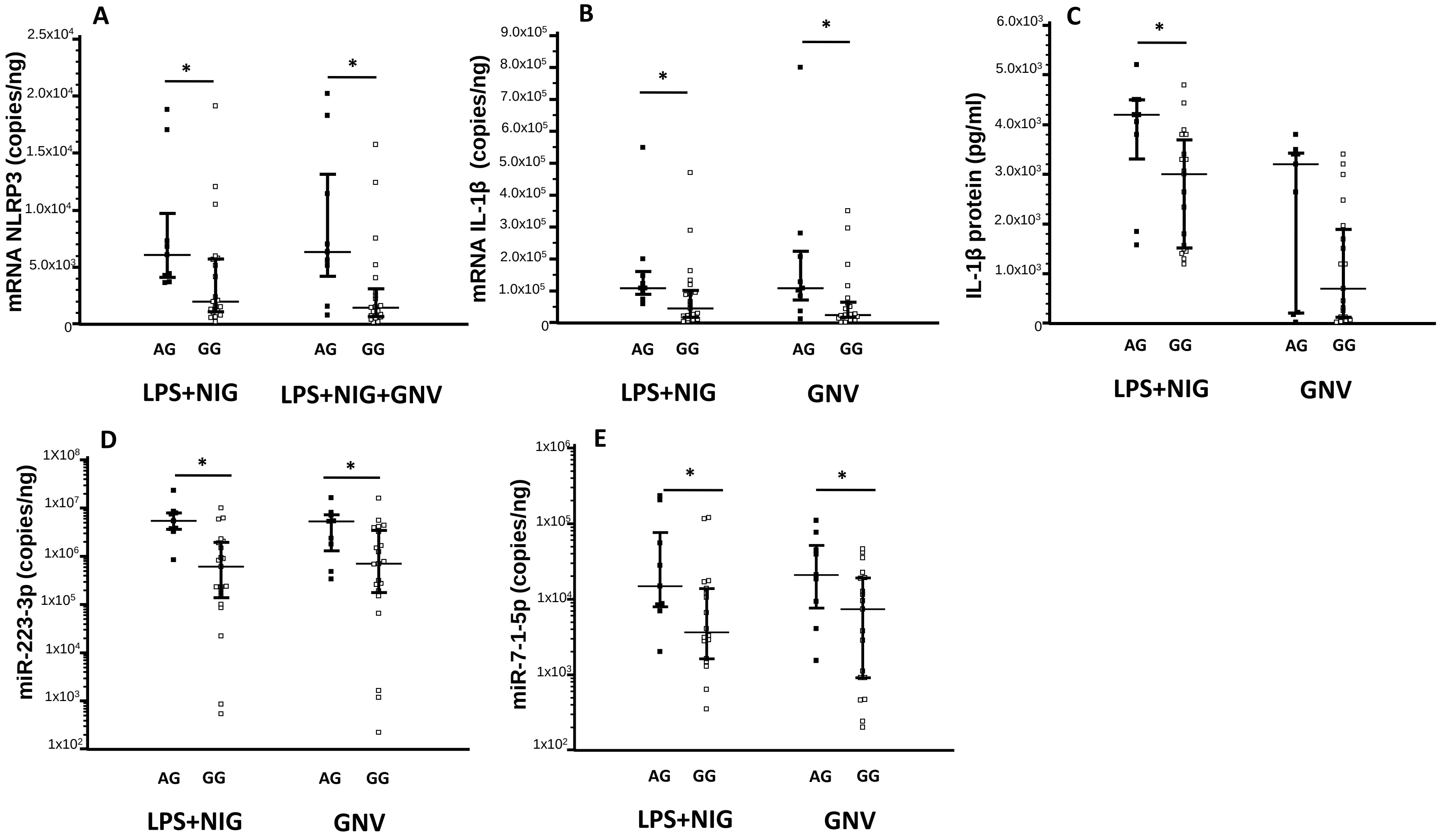

Supplement: Supplementary file 1 [file biomolecules-15-01074-s001.zip › biomolecules-3738992-supplementary.pdf]
